# Detection of HPV in oral rinse samples from OPSCC and non-OPSCC patients

**DOI:** 10.1186/s12903-015-0111-x

**Published:** 2015-10-15

**Authors:** Juliet Dang, Qinghua Feng, Keith D. Eaton, Hona Jang, Nancy B. Kiviat

**Affiliations:** Oral Health Sciences, University of Washington, Box 357475, 1959 NE Pacific St., Seattle, WA 98195 USA; Seattle Cancer Care Alliance, 825 Eastlake Ave E, Seattle, WA 98109 USA; Pathology, University of Washington, Seattle, WA USA; Harborview Medical Center, 908 Jefferson, Seattle, WA 98104 USA; University of Washington, 1959 NE Pacific St., Seattle, WA 98195 USA

## Abstract

**Background:**

Due to the increasing rates of oropharyngeal cancer, oral HPV infection is a significant concern. Methods for detecting oral HPVs is not standardized as there are different techniques available. We propose that use of oral rinse samples to detect for HPVs is a suitable technique within a clinic setting. Thus, our main objective is to study HPV detection in oral rinse samples.

**Methods:**

In our study, we used oral rinse sample collection coupled with real-time PCR to detect for HPVs types 16 and 18, and preferentially amplified FAP PCR samples to detect for a broad range of HPVs, in oropharyngeal squamous cell carcinoma (OPSCC), non-OPSCC, and healthy patients.

**Results:**

Thirty three percent of 100 cancer patients were positive for any type of HPV; of those 23 were positive for HPV16. Only 1 of 110 healthy controls was positive (this subject was positive for HPV18).

**Conclusion:**

Our results indicate that HPV detection in oral rinse samples may be useful as a screening tool to detect HPV-associated oral cancers.

## Background

Tissues infected by human papillomavirus (HPV) have the ability to evolve into an HPV-associated cancer. This is likely due to a field effect where HPV can modify regular cell functions resulting in malignancy. Though we know how to test for HPV-related cancers, there is currently no standard for detecting HPV infection [1], and screening for HPV could identify individuals at risk for head and neck squamous cell carcinoma (HNSCC). Speaking of which, HNSCC cases associated with HPV have been a challenge to screen for, especially oral cavity squamous cell carcinoma (OSCC) and oropharyngeal squamous cell carcinoma (OPSCC) cases, which are subsets of HNSCC. The oropharyngeal area includes the base of the tongue, soft palate, tonsils, and tonsillar region, with the oral cavity encompassing the rest of the interior tissues of the mouth. The most common methods for HPV detection within the mouth and oropharynx begin with collection of cells with a cotton swab, cytobrush, or a mouth rinse [2], followed by the use of PCR-based assays or DNA in situ hybridization [3]. However, there are challenges present for certain techniques. For example, the use of a swab/brush limits the amount of mucosa that is sampled, and obtaining a sample from a non-visible lesion within the tonsillar crypt may not be feasible [4]. The base of the tongue is not entirely accessible either as there is both flat mucosa and tonsillar tissue, thus increasing the risk for false negatives [5]. We chose to use a mouth rinse technique for sample collection as it is non-invasive, quick, and simple for the patient.

Analyzing p16 expression has been used as a biomarker for HPV-associated OSCC/OPSCC, but studies have reported that p16 overexpression is not always present in cases involving oncogenic HPVs [6–9]. A recent study concluded that p16 should not be used as a surrogate marker for HPV infection in oral cancers due to poor concordance between the two [10]. Previous to this, Pannone et al. also stated that p16 immunohistochemistry (IHC) alone does not prove to be a reliable method in HPV detection for OSCC/OPSCC cases [11]. Within our study, we obtained information for p16 testing to see whether or not our HPV data was in concordance.

OPSCC incidence in developed countries has increased significantly and HPV infection is proposed to be the main factor [12]. Risk factors for oral HPV infection include certain sexual practices [13–16]; number of lifetime partners and number of recent sex partners, older age, being male, and current cigarette smoking [17, 18]. The most prevalent type of HPV associated with oral infection is type 16 [17, 19], which has been demonstrated to be oncogenic in HNSCCs [20]. A worldwide systematic review of HNSCC biopsies demonstrated HPV16 in 31 % of OPSCC cases; 16 % of OSCC cases; and 17 % of laryngeal SCC cases [21]. HPV type 18 also appears to play a significant role in carcinogenesis, especially in the oropharynx [22, 23].

With the rise of OPSCCs it is imperative to have a gold-standard technique in place for oral HPV detection. Collecting samples from the oral cavity and oropharynx for the detection of oral HPVs should be quick, non-invasive, inexpensive, and sufficient in HPV DNA collection. In our study, we investigated oral rinse samples coupled with real-time PCR Taqman assays to detect for HPV types 16 and 18, which is sensitive and specific. To detect for a broad range of HPVs, we preferentially amplified the oral rinse samples from cancer cases, and used fluorescent arbitrarily primed (FAP) PCR, a general PCR method using degenerate HPV primers.

### Study population

Between 2011 and 2013, we recruited 76 OPSCC and 24 non-OPSCC patients from the Seattle Cancer Care Alliance (Seattle, WA), and 110 healthy subjects from University of Washington Dental Clinic (Seattle, WA). Non-OPSCC cases included patients with OSCC, laryngeal, sinus, and supraglottis cancers. We screened the schedules of five oncologists in order to identify eligible cancer patients, and discussed our study at their appointment. One patient declined due to mouth sores and sensitivity. 21/100 cancer patients had already begun treatment and of these 21 patients: 18 patients had treatment less than 21 days before sampling, two had over 30 days of treatment, and one patient had treatment for 7 months. Healthy subjects were randomly selected within the student dental clinic, one patient declined to take part in the study. Inclusion requirements for the healthy population included being: cancer-free, not pregnant, HIV-free, and over the age of 16. Each patient signed written consent forms to participate in the study (IRB #7490 approved April 9, 2014), and answered a simple health questionnaire. Gender, age, race, smoking, alcohol, and marijuana history were recorded for all subjects. Charts for a subset of our patients were reviewed for p16 immunohistochemistry (IHC) and HPV testing.

## Methods

### Collection & DNA purification methods

For sample collection, all patients rinsed and gargled for 30 s with Original Mint Scope® mouthwash (Procter & Gamble). Four normal healthy individuals requested to use Crest® Alcohol-free mouthwash due to a history of alcoholism. Oral rinse samples were centrifuged for 15 min. at 4 °C to form a pellet, the supernatant was discarded, and the pellet was placed in -80 °C until further processing. The Puregene® DNA Purification Kit was used to isolate genomic DNA from the buccal cell pellet within the mouthwash samples (Qiagen item #158467, manufacturer’s protocol was followed).

All human subjects IRB protocols and regulations were followed under the Fred Hutchinson Cancer Research Center guidelines (IRB #7490 approved April 9, 2014).

### HPV & analytic methods

Taqman real-time PCR assays were used for detection on the ABI Prism 7900 Sequence Detection System with 40 cycles in a reaction (denaturation at 95 °C, annealing and extension at 60 °C). Absolute quantification was used to determine HPV16 and 18 viral load, and total human genomic DNA in the sample was determined on Alu sequences. Serial dilutions of human genomic DNA and full length HPV16 and 18 plasmids, of known concentrations, were used as standard curves.

- HPV16 E7 Primers
- Forward: CGGACAGAGCCCATTACAATATT
- Reverse: CGCACAACCGAAGCGTAGA
- HPV16 E7 Probe: TAACCTT(T/C)TGTTGCAAGTGT
- HPV18 E7 Primers:
- Forward: CCGACGAGCCGAACCA
- Reverse: TGGCTTCACACTTACAACACATACA
- HPV18 E7 Probe: AACGTCACACAATGTT

In order to increase the efficiency of HPV detection, we used the multiply-primed rolling-circular amplification technique (MP-RCA) to preferentially amplify unknown, circular HPV DNA. MP-RCA has been demonstrated to amplify circular DNA templates up to 107-fold [24]. The TempliPhi 100 Amplification Kit (Amersham Biosciences) protocol was followed.

**Fig. 1:**
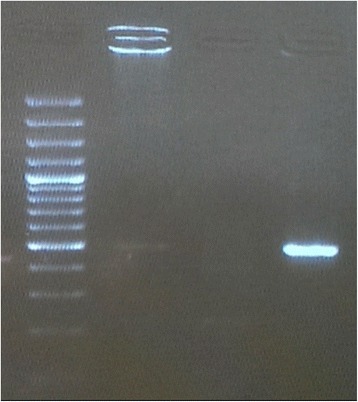
FAP PCR results on an electrophoresis gel. An expected band of ~480 bp indicates an HPV positive sample

The FAP PCR published protocol was followed to detect for a broad range of HPV types where primers were developed from conserved L1 regions [25]. We only performed this technique on the cancer case samples (Fig. 1).

We categorized smoking history as follows: non-smoker (0 packs); light smoker (<1pack/week); moderate smoker (≥1pack/week ≤1 pack/day); heavy smoker (≥1pack/day). For those who smoked cigars or chewed tobacco we calculated to the equivalent of packages of cigarettes smoked. Alcohol history was categorized as follows: none (never drinks); rarely/occasionally (1 drink every 1–2 months); light (1–6 drinks/week for females, 1–13 drinks/week for males); moderate (7 drinks/week for females, 14 drinks/week for males); heavy (>7drinks/week for females, >14 drinks/week for males).

All data analysis was done using Stata MP 13.1 (StataCorp LP, Texas, USA).

## Results

Oral cancer subjects with OPSCC and non-OPSCC were more likely to be male than control subjects without cancer, as at least 83 % of subjects in each cancer type were male, compared to only 46 % of controls (Table 1; *p* <0.001). Cancer cases were also older than controls, as >80 % of cases, regardless of cancer type, were 50 years of age or older compared to only 63 % of controls (*p* <0.001). Subjects with OPSCC and non-OPSCC were similar to subjects without cancer with respect to race (*p* = 0.30) and ethnicity (*p* = 0.62) and were predominately non-Hispanic Caucasians. Those who had cancer (39 % OPSCC, 54 % non-OPSCC; *p* <0.001) were more likely to be heavy smokers compared to those with no cancer (32 %). Heavy alcohol history was prevalent in 33 % of OPSCC and in 33 % of OSCC patients (*p* = 0.015) compared to only 12 % of non-cancer subjects. Marijuana use was mainly observed in cancer cases (11 % OPSCC, 4 % OSCC; *p* = 0.010) and rarely in controls (0.91 %). Cases of both cancer types (33 % OPSCC, 33 % non-OPSCC; *p* <0.001) were more likely to have detectable HPV than healthy controls (0.9 %), and specifically type 16 (25 % OPSCC, 17 % non-OPSCC; *p* <0.001) detected in their oral rinse samples compared to controls. HPV18 detection did not vary by study group and was detected in a single sample from a control subject (*p* = 0.63), and none in the cancer cases. Our method for HPV detection had a sensitivity of 33 % and specificity of 99 % (*p* <0.001).

**able 1:** Demographics amongst all subjects in non-cancer and cancer cases

	No cancer	OPSCC	Non-OPSCC	*p*-value
	*n* = 110	*n* = 76	*n* = 24	
Gender				
Male	51 (46.36 %)	63 (82.89 %)	20 (83.33 %)	<0.001
Female	59 (53.64 %)	13 (17.11 %)	4 (16.67 %)	
Grouped age				
20–39	27 (24.55 %)	1 (1.32 %)	1 (4.17 %)	
40–49	13 (11.82 %)	10 (13.16 %)	2 (8.33 %)	
50–59	21 (19.09 %)	29 (38.16 %)	11 (45.83 %)	<0.001
60–69	29 (26.36 %)	30 (39.47 %)	8 (33.33 %)	
70+	20 (18.18 %)	6 (7.89 %)	2 (8.3 %)	
Race				
Asian	5 (5.55 %)	2 (2.63 %)	1 (4.17)	
Black	6 (5.45 %)	1 (1.32 %)	0	
White	92 (83.64 %)	65 (85.53 %)	21 (87.50 %)	0.30
Other	7 (6.36 %)	4 (5.26 %)	1 (4.17 %)	
Unknown	0	4 (5.26 %)	1 (4.17 %)	
Ethnicity (Hispanic/Latino)				
Yes	4 (3.64 %)	2 (2.63 %)	0	0.62
No	106 (96.36 %)	74 (97.37 %)	24 (100 %)	
Smoking history				
Non-smoker	51 (46.36 %)	21 (27.63 %)	5 (20.83 %)	
Light smoker	1 (0.91 %)	14 (18.42 %)	2 (8.33 %)	<0.001
Moderate smoker	23 (20.91 %)	11 (14.47 %)	4 (16.67 %)	
Heavy smoker	35 (31.82 %)	30 (39.47 %)	13 (54.17 %)	
Alcohol history				
None	24 (21.82 %)	7 (9.21 %)	3 (12.50 %)	
Rarely/occasionally	16 (14.55 %)	14 (18.42 %)	4 (16.67 %)	
Light	54 (49.09 %)	27 (35.53 %)	9 (37.50 %)	0.015
Moderate	3 (2.73 %)	3 (3.95 %)	0	
Heavy	13 (11.82 %)	25 (32.89 %)	8 (33.33 %)	
Any Marijuana use				
Yes	1 (0.91 %)	8 (10.53 %)	1 (4.17 %)	0.010
No	109 (99.09 %)	68 (89.47 %)	23 (95.83 %)	
Any HPV	1 (0.91 %)	25 (32.89 %)	8 (33.33 %)	<0.001
HPV 16 positive	0	19 (25.00 %)	4 (16.67 %)	<0.001
HPV 18 positive	1 (0.91 %)	0	0	0.63

^a^Data for “No Cancer” is associated with published data from *Prevalence of HPV types 16 and 18 within a dental student clinic setting* (J Dang et al.)

Twenty-two HPV positive samples were tested for p16 prior to sampling, and only one tested negative for p16. However, 26 samples that were negative for HPV were positive for p16 (Table 2). It should be noted that three of the 26 patients (*p* = 0.085) underwent treatment (ie. chemotherapy, radiation therapy, excision) prior to sampling, which could affect the presence of prior HPV infection. HPV was marginally associated with p16 detection (*p* = 0.076); of patients who were HPV positive, 96 % had a positive p16 test; only one sample that was positive for HPV tested negative for p16. Amongst HPV negative patients, 79 % were p16 positive. Only two case patients had HPV screening completed before sampling and both were negative for HPV in our tests and the screening.

**Table 2:** p16 and any HPV

Any HPV	p16 test		*p*-value
	Positive	Negative	0.076
	*n* = 48	*n* = 8	
Yes	22 (95.65 %)	1 (4.35 %)	
No	26 (78.79 %)	7 (21.21 %)	

## Discussion

Patient sample collection can be a difficult and time consuming task especially when the researcher is interrupting an appointment. Also, many individuals have sensitive gag reflexes, thus if a brush or swab technique is used to scrape the back of the throat, obtaining a sample may be quite difficult without the aid of a topical anesthetic. This is why it is imperative to have a sampling technique in place that is non-invasive and quick, yet sufficient for detection of HPV. Our study has demonstrated that oral rinse sample collection is an unobtrusive method to use for detection of oral HPVs. This is in concordance with a study comparing oral rinse and cytology brush sampling, which concluded that oral rinses were the best choice for sampling from the oral cavity in order to detect for HPV [26].

We used Scope® because it was observed to have excellent preservation of high-molecular-weight DNA quality, and it is more palatable than most other rinse media [27]. Four control patients requested to rinse with Crest Pro-Health® non-alcoholic mouthwash. We did not see differences in DNA quantity (data not shown), however more research is required to see if there is a difference with quantity as well as quality.

**Fig. 2:**
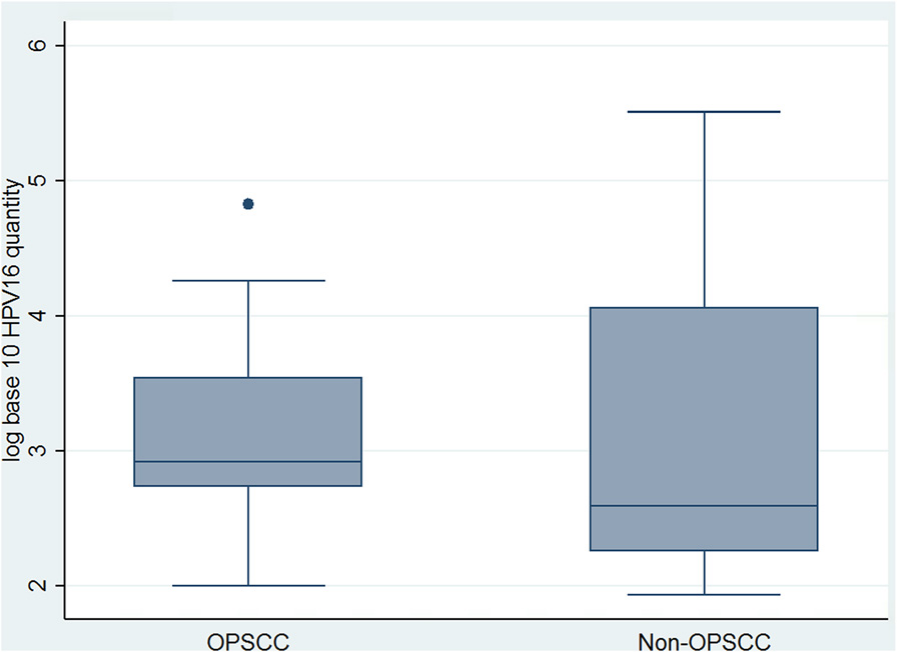
Comparison of HPV16 viral load (copies/ng DNA) between the two cancer groups. No statistically significant difference was seen (*p* = 0.40). The gray boxes demonstrate range from lower to upper quartiles. The median is represented by a horizontal line. Maximum and minimum values are indicated by the vertical lines. Viral load variation between each group was demonstrated. The median viral load in OPSCC was slightly higher than non-OPSCC cases

Quantifying viral load may be crucial in determining whether HPV-positive OPSCC/OSCCs are unquestionably the result of HPV infection [28]. The gold-standard for HPV viral load assessment is real-time PCR [29, 30]. One study demonstrated OPSCC to have a viral load of ~80,000 times higher than OSCC and other HNSCCs [29]. Our results showed no significant differences between the two cancer groups (Fig. 2). As well, differences in results could be due to the authors using fresh-frozen biopsies where only a small portion of the oral cavity or oropharynx is sampled. Oral rinse samples may allow for a more effective collection of oral cells for HPV detection. Variation in findings warrants further investigation of viral load in association with subsets of HNSCC.

The one patient with OPSCC, who had undergone treatment for 7 months prior to sampling, was positive for HPV, specifically type 16. This case is unique as undergoing several months of treatment should eradicate the virus unless if the infection was recent. It should be noted that the patient had a heavy smoking and drinking history. More research on prevalence of HPV infection after radiation/chemotherapy is warranted.

One major limitation was not having DNA from tumor tissue to compare HPV detection. The tissue samples were not available to us, but would have acted as a gold standard within our study. Other limitations to our study include: our results demonstrating a low sensitivity for HPV detection in cancer patients, but with a very high specificity; we could not control how well a patient would gargle and swish the mouthwash, which will have an effect on the quantity and quality of DNA collected; Scope® has a strong mint taste, which may not be suitable for those with sensitive mouths; small sample size; not detecting for HPV RNA to show active infection; and FAP PCR is sensitive enough to detect for a broad range of HPVs, but some HPVs may not be detected due to the generality of the designed primers [25].

p16 testing is usually only performed in cases where they do not fit the traditional risk factors (ie. significant tobacco and alcohol use history), and HPV testing is rarely done. This is something that needs to be changed during diagnosis due to the increase in HPV-associated OPSCCs.

## Conclusions

From our results we demonstrated that preferentially amplified oral rinse samples with HPV detection from real-time PCR Taqman assays and FAP PCR, is a usable method overall, which could be used within a clinic setting. More specific studies will need to be done to determine whether mouth rinses do actually reflect, in cases, the type of HPV associated with the cancerous lesion.

## Abbreviations

OSCC: Oral squamous cell carcinoma
OPSCC: Oropharyngeal squamous cell carcinoma
HNSCC: Head and neck squamous cell carcinoma
RT-PCR: Real-time polymerase chain reaction
FAP PCR: Fluorescent arbitrarily primed polymerase chain reaction
MP-RCA: Multiply-primed rolling-circular amplification
